# A mouse line for inducible and reversible silencing of specific neurons

**DOI:** 10.1186/s13041-014-0068-8

**Published:** 2014-09-18

**Authors:** Ling Hu, Wei Lan, Hao Guo, Guo-Dong Chai, Kun Huang, Ling Zhang, Ying Huang, Xue-Feng Chen, Lei Zhang, Ning-Ning Song, Ling Chen, Bing Lang, Yun Wang, Qing-Xiu Wang, Jin-Bao Zhang, Collin McCaig, Lin Xu, Yu-Qiang Ding

**Affiliations:** Key Laboratory of Arrhythmias, Ministry of Education of China, East Hospital, Tongji University School of Medicine, Shanghai, 200120 China; Department of Anatomy and Neurobiology, Tongji University School of Medicine, Shanghai, 200092 China; School of Medical Sciences, Institute of Medical Sciences, University of Aberdeen, Foresterhill, Aberdeen, AB25 2ZD UK; Key Laboratory of Animal Models and Human Disease Mechanisms of the Chinese Academy of Sciences and Yunnan Province, Kunming Institute of Zoology, Kunming, Yunnan 650223 China; Department of Anesthesiology, East Hospital, Tongji University School of Medicine, Shanghai, 200120 China; Institutes of Brain Science, State Key Laboratory for Medical Neurobiology, Fudan University, Shanghai, 200032 China; Institute of Neuroscience, School of Basic Medical Sciences, Wenzhou Medical University, Wenzhou, Zhejiang 325035 China

**Keywords:** Neuron silencing, Ligand-gated channel, Ivermectin, Rosa26

## Abstract

**Background:**

Genetic methods for inducibly and reversibly inhibiting neuronal activity of specific neurons are critical for exploring the functions of neuronal circuits. The engineered human glycine receptor, called ivermectin (IVM)-gated silencing receptor (IVMR), has been shown to possess this ability *in vitro*.

**Results:**

Here we generated a mouse line, in which the IVMR coding sequence was inserted into the ROSA26 locus downstream of a loxP-flanked STOP cassette. Specific Cre-mediated IVMR expression was revealed by mis-expression of Cre in the striatum and by crossing with several Cre lines. Behavioral alteration was observed in Rosa26-IVMR mice with unilateral striatal Cre expression after systemic administration of IVM, and it could be re-initiated when IVM was applied again. A dramatic reduction in neuron firing was recorded in IVM-treated free moving Rosa26-IVMR;Emx1-Cre mice, and neuronal excitability was reduced within minutes as shown by recording in brain slice.

**Conclusion:**

This Rosa26-IVMR mouse line provides a powerful tool for exploring selective circuit functions in freely behaving mice.

**Electronic supplementary material:**

The online version of this article (doi:10.1186/s13041-014-0068-8) contains supplementary material, which is available to authorized users.

## Background

Understanding causal links between the activity of specific neuronal circuits and behavior is critical and challenging. Numerous efforts have been made to achieve this, and various tools have been developed to perturb circuit function, such as Molecular System for the Inactivation of Synaptic Transmission, optogenetics (e.g. rhodopsin/halorhodopsin) and ligand-gated ion channels [1–4].

Several ligand-gated channels have been developed to inactivate defined neuronal population in an inducible and reversible way. The *Drosophila* allatostatin receptor, which suppresses action potential generation upon selective activation by allatostatin [5], has been used successfully to inactivate neuronal activity in mammalian brain [6–8]. However, because allatostatin cannot cross the blood-brain barrier, it must be delivered locally by an invasive method and is inconvenient when a large number of animals are used in behavioral experiment. Recently, an engineered GABA_A_ receptor, which is insensitive to zolpidem, has been used for inducibly silencing specific neurons [9]. One limitation of this system is that it cannot be used in neurons without expressing GABA_A_ γ2 subunit [1]. The third system of reducing neuronal firing is the IVM-gated chloride channel from *C. elegans* GluCl α and β subunits, and this works well in mice as shown by amphetamine-induced rotational behavior [10,11]. The potential limitation of this method is the requirement of expressing the two different subunits in the same neuronal population, which may limit its use. To overcome this, the human α1 glycine receptor was modified by mutation of F207A and A288G, named as IVMR, which is insensitive to glycine but sensitive to IVM, and able to reduce neuronal excitability *in vitro* [12], therefore offering a new tool for inducible and reversible silencing neurons.

In this study, the IVMR coding sequence was knocked into Rosa26 locus downstream of a loxP-flanked STOP cassette. Specific Cre-mediated IVMR expression was verified by *in situ* hybridization and immunostaining, and inducible and reversible inactivation of neuronal activity was revealed by two behavioral observations. Reduction of neuronal firing was recorded in the hippocampus of free moving Rosa26-IVMR;Emx1-Cre mice, and data from brain slice suggested that this reduction is caused by reduced neuronal excitability.

## Results

### Morphological characterization of Rosa26-IVMR mice

**Figure 1:**
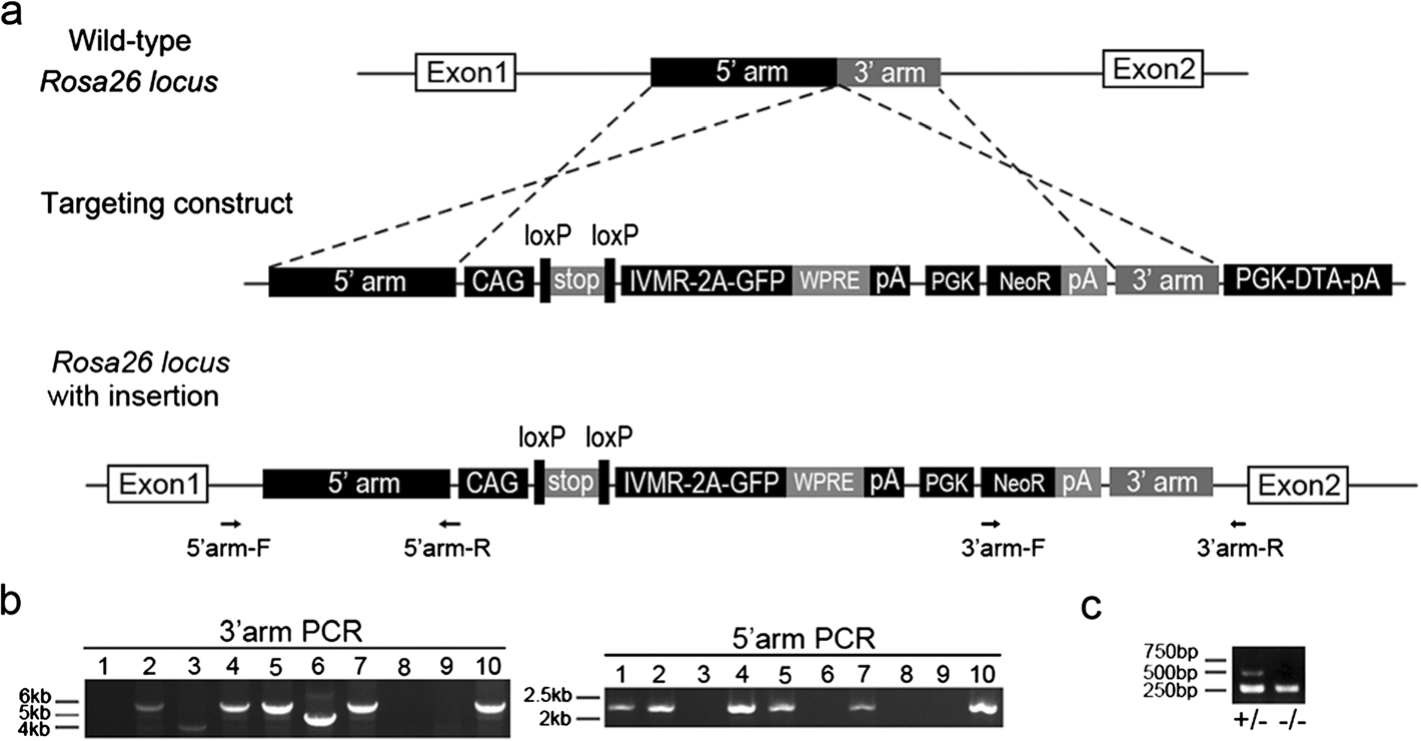
**Gene targeting of the IVMR-2A-GFP sequence into the ROSA26 locus by homologous recombination. (a)** Diagram of the targeting construct and the expected recombination events. CAG, CAG promoter; WPRE, the woodchuck hepatitis virus posttranscriptional regulatory element; pA, polyA sequence; PGK, PGK promoter; NeoR, neomycin resistance positive selection cassette; DTA, diphtheria toxin negative selection cassette. **(b)** 5′arm and 3′arm PCR analysis showing the identification of positive targeted ES clones (Lanes 2, 4, 5, 7 and 10). **(c)** Genotyping of mouse pups to identify offspring harboring the conditional allele.

**Figure 2:**
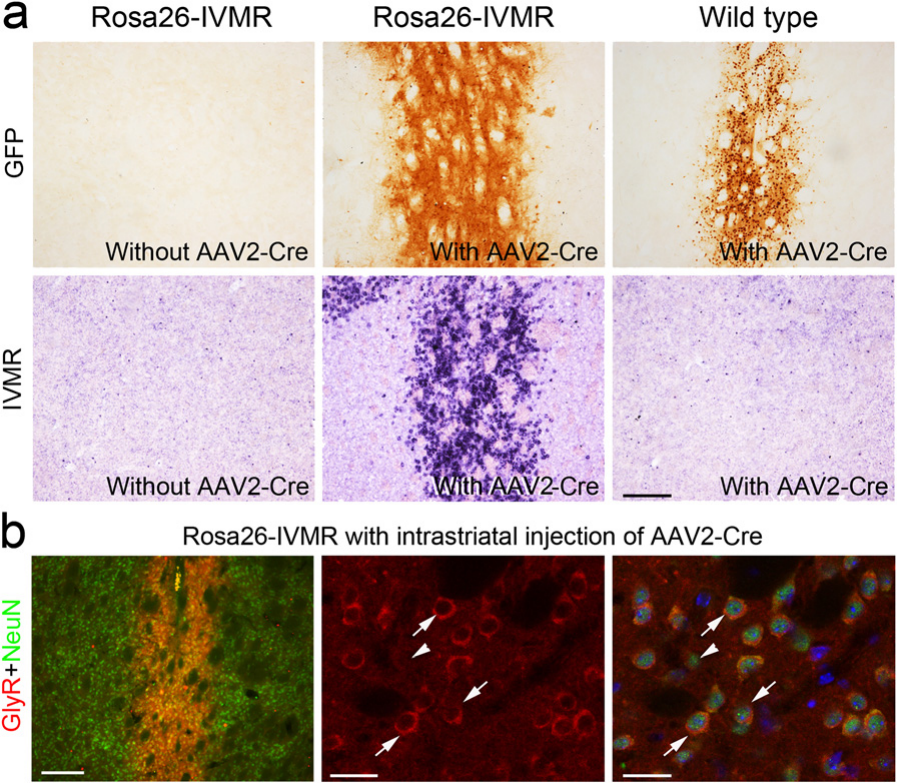
**AAV2-Cre-mediated IVMR expression in the striatum of Rosa26-IVMR mice.** Mice were sacrificed 14 d after injection of AAV2-Cre virus. **(a)** IVMR expression shown by GFP immunostaining and in situ hybridization is present in Rosa26-IVMR mice, but not in Rosa26-IVMR mice without AAV2-Cre or in wild type mice with AAV2-Cre injection. AAV2-Cre virus also expresses GFP. **(b)** Human glycine receptor immunoreactivity (red) is located in NeuN-immunopositive (green) neurons in the striatum of Rosa26-IVMR mice. Arrows point to NeuN/IVMR-double positive neurons, and Hoechest (blue) indicates nuclei. Scale bars, 200 μm in (a) and left panel in (b), and 40 μm in the other panels in (b).

**Figure 3:**
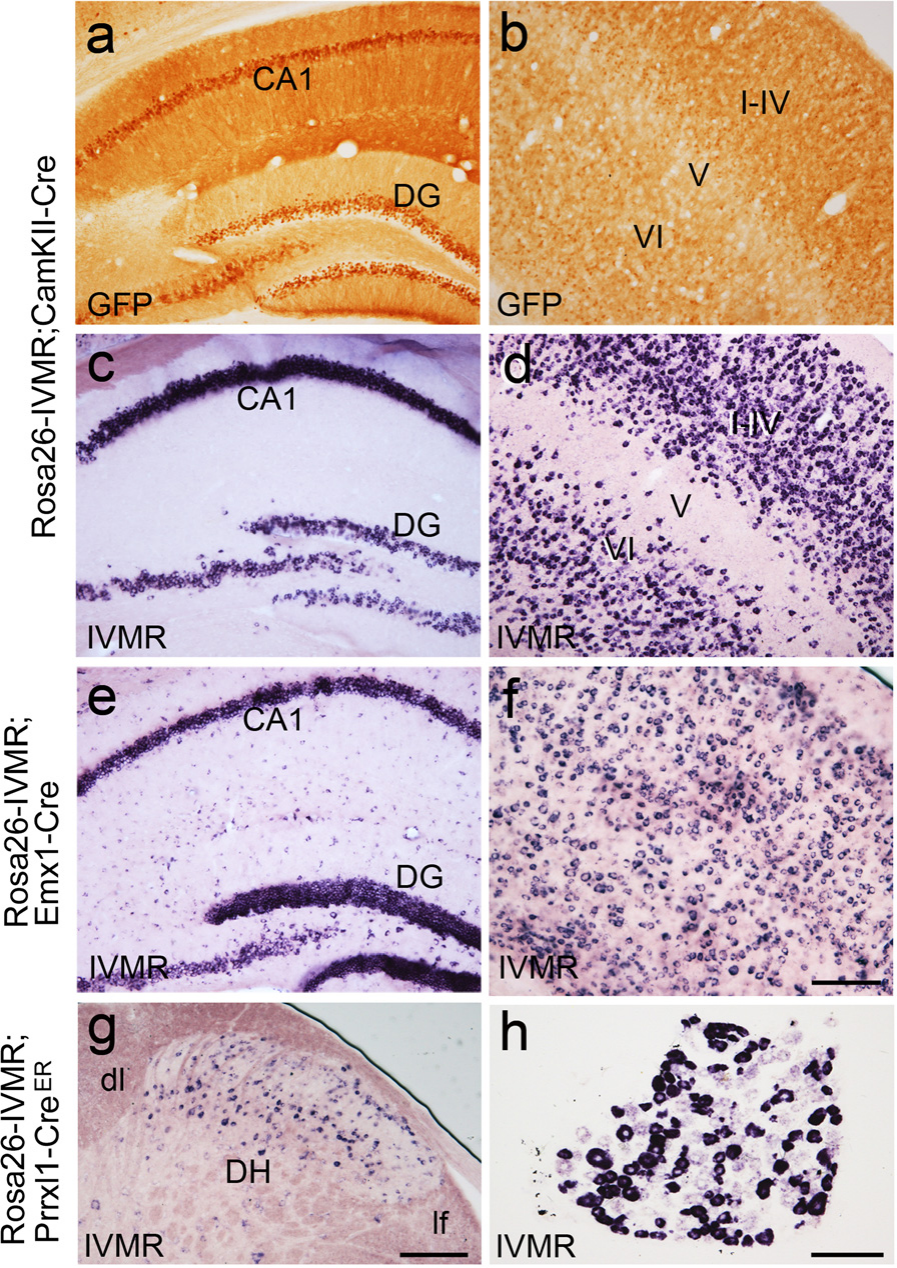
**IVMR expression in Rosa26-IVMR mice crossed with 3 Cre lines. (a-d)** GFP immunostaining and IVMR in situ hybridization in the hippocampus (a, c) and cerebral cortex (b, d) of Rosa26-IVMR;CamKII-Cre mice. **(e, f)** Expression of IVMR mRNA in the hippocampus (e) and cerebral cortex (f) of Rosa26-IVMR;Emx1-Cre mice. **(g, h)** Expression of IVMR mRNA in the spinal dorsal horn (g) and dorsal root ganglion (h) of tamoxifen-treated Rosa26-IVMR;Prrxl1-Cre^ER^ mice. CA1, CA1 region of the hippocampus; df, dorsal fasciculus; DG, dentate gyrus; DH, dorsal horn; lf, lateral fasciculus; I-VI, cortical layers. Scale bars, 200 μm in (f) applies to (a-e), 150 μm in (g) and 100 μm in (h).

Rosa26-IVMR mice were generated by knocking IVMR-2A-GFP coding sequence into the Rosa26 locus (Figure 1). Homozygous and heterozygous Rosa26-IVMR mice were viable, fertile, normal in size, and did not display any gross physical abnormalities. Cre-mediated IVMR expression was revealed by *in situ* hybridization for IVMR mRNA. As shown in Figure 2a, injection of AAV2-Cre virus into the striatum resulted in local IVMR expression in Rosa26-IVMR mice but not wild type mice although Cre was misexpressed (Additional file 1: Figure S1). Immnostaining of the human glycine receptor showed intense immunoreactivity within the neurons of the virus-injected area (Figure 2b). In addition, Rosa26-IVMR mice were crossed with several Cre lines to further confirm Cre-mediated IVMR expression. In Rosa26-IVMR;CamKII-Cre mice, IVMR mRNA was abundantly distributed in the cerebral cortex, hippocampus and thalamus (Figure 3b,c), which is consistent with the Cre expression pattern in CamKII-Cre mice [13]. With the help of 2A-peptide self-processing system (Figure 1), which ensures faithful expression of multiple proteins [14], GFP could be used to detect IVMR expression. The expression pattern of GFP immunoreactivity was very similar to that of IVMR mRNA, although it was localized also in dendrites in addition to cell bodies (Figure 3a,b). In addition, Cre-dependent IVMR expression was observed in Rosa26-IVMR;Emx1-Cre mice, which was abundantly distributed in the cerebral cortex and hippocampus (Figure 3e,f), consistent with Cre expression pattern of Emx1-Cre mice [15]. However, no IVMR mRNA or GFP immunoreactivity was detected in Rosa26-IVMR mice without Cre (Figure 1a). Taken together, we have shown that Cre-dependent IVMR expression can be achieved in Rosa26-IVMR mice allowing IVMR expression in specific cell-type neurons.

### Functional characterization of Rosa26-IVMR mice

Unilateral disruption of the nigrostriatal pathway is widely used in studying movement disorders, and animals with unilateral damage to the striatum show rotation towards the lesioned side in the presence of amphetamine or apomorphine [11,16]. Apomorphine-induced rotation therefore can be used at a behavioral level to explore the efficacy of genetic tool in silencing neuronal activity. The mice were placed into 40 × 40 cm open field box (Med Associate, USA), their locomotion was videotaped, and rotation behaviors were analyzed 5 min after administration of apomorphine using Ethovision XT8 software (Noldus Inc., The Netherland). Full 360° rotations without any changes in direction were counted for 20 min. Rotation scores were calculated by subtracting contralateral from ipsilateral rotations and dividing by the total travel distance.

**Figure 4:**
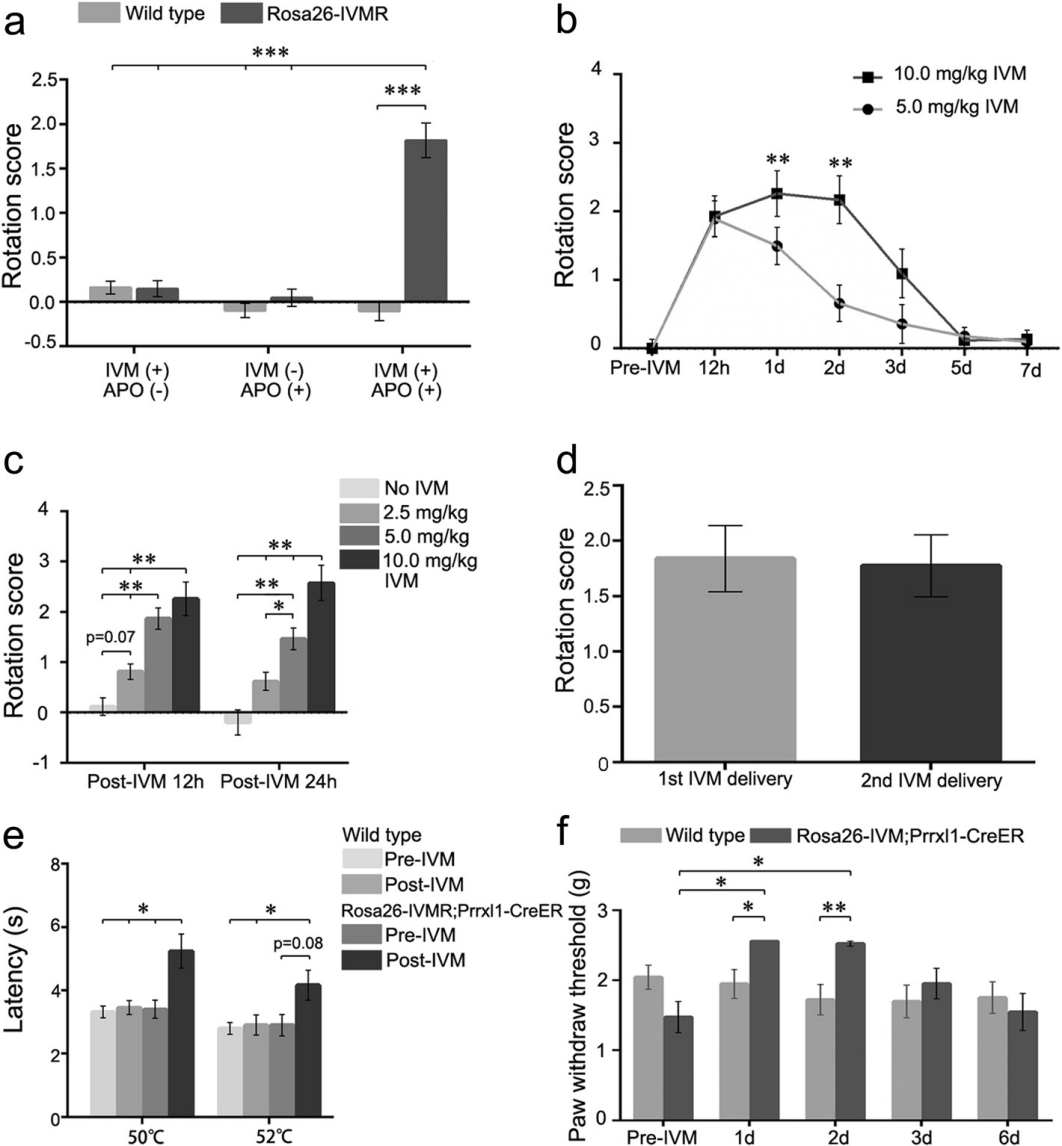
**IVM-induced behavioral alteration in free moving Rosa26-IVMR mice. (a)** Rotation towards AAV2-Cre virus-injected side is only present in Rosa26-IVMR mice treated with both apomorphine and IVM but not those treated with one of them. Wild type controls do not show any rotation when treated with both of them or either of them. Apomorphine-induced rotation was performed 12 h after administration of IVM (10 mg/kg). n = 6 for wild type; n = 8 for Rosa26-IVMR mice; ***P < 0.001, two-way repeated ANOVA test. **(b)** Time course of apomorphine-induced rotation in Rosa26-IVMR mice with unilateral injection of AAV2-Cre virus. n = 9 for each; **P < 0.01 (5 mg/kg vs 10 mg/kg group), two-way repeated ANOVA test. **(c)** Dose effect of IVM on apomorphine-induced rotation in Rosa26-IVMR mice at 12 h and 1 d post systemic administration. Six to eleven mice were included in each set of experiments; **P < 0.01, one-way ANOVA test. **(d)** Apomorphine-induced rotation is similar between the first- and second cycles of IVM administration. This observation was done 1 d after IVM treatment (10 mg/kg). n = 6 for each; P = 0.7, paired samples test. **(e)** Thermal sensation shown by the tail immersion test is lowered in Rosa26-IVMR;Prrxl1-Cre^ER^ mice 1 d after administration of IVM (10 mg/kg), but unchanged in wild type mice. n = 11 for wild type, n = 13 for Rosa26-IVMR;Prrxl1-Cre^ER^ mice; *P < 0.05, two-way repeated ANOVA test. **(f)** Mechanical sensation shown by the von Frey test is decreased in Rosa26-IVMR;Prrxl1-Cre^ER^ mice at 1 d and 2 d after systemic administration of IVM, but unchanged in wild type mice. n = 11 for wild type, n = 13 for Rosa26-IVMR;Prrxl1-Cre^ER^ mice; *P < 0.05, **P < 0.01, two-way repeated ANOVA test.

In Rosa26-IVMR mice with unilateral injection of the AAV2-Cre virus, administration of apomorphine resulted in an obvious rotation towards the injection side 12 h after a single intraperitoneal administration of IVM at 10 mg/kg (Figure 4a). No rotation behaviors were found in AAV2-Cre virus-injected Rosa26-IVMR mice without IVM or apomorphine treatment, or in Rosa26-IVMR mice without AAV2-Cre virus injection, or in wild type control (Figure 4a). Thus, systemic administration of IVM is able to activate IVMR of striatal neurons leading to behavioral alteration.

We then examined rotation behaviors at later time points. As shown in Figure 4b, apomorphine-induced rotation reached peak level at 1 d and 2 d, reduced at 3 d and disappeared at 5 d and 7 d. We next explored dose responses of IVM. IVM at 5 mg/kg also led to obvious rotation behaviors, but the time window was narrowed to a 12 h-1d period (Figure 4b). When IVM was applied at 2.5 mg/kg, no obvious rotation behaviors were found relative to those at 5 and 10 mg/kg (Figure 4c). Because 5 mg/kg of IVM induced similar rotation behaviors at 12 h relative to 10 mg/kg, we used this concentration to examine the earliest onset of apomorphine-induced rotation. Clear rotation behavior was detected at 9 h after systemic administration of IVM, but rotation scales varied greatly among animals (Additional file 2: Figure S2); this may reflect insufficient neuron silencing because IVM has not reach required concentration for activation of IVMR. Nevertheless, IVM is able to inhibit the activity of striatal neurons of Rosa26-IVMR mice in a reversible way and this effect is IVM dose-dependent.

We next set out to explore if the striatal neurons can be re-inactivated with IVM in the same mice. Mice were treated with IVM (10 mg/kg) again 3 weeks after the initial IVM administration (10 mg/kg). Apomorphine-induced rotation was examined at 1 d, and rotation score was not different from that obtained in the initial IVM administration (Figure 4d). These results indicate that IVMR maintains its sensitivity to IVM in mouse brain offering the availability of multiple cycles of neuron silencing.

The silencing effect by activation of IVMR was examined also in Rosa26-IVMR;Prrxl1-Cre^ER^ mice [17], in which Cre-mediated IVMR expression was induced by tamoxifen treatment in the sensory neurons located in the dorsal root ganglia and spinal dorsal horn (Figure 3g, h). Transcription factor *Prrxl1* is selectively expressed in the somatosensory system and involved in the somatosensory sensation [18–20]. The tail immersion test was used to examine thermal sensation 1 d after systemic administration of IVM. Tail flick latency was largely increased in IVM-treated Rosa26-IVMR;Prrxl1-Cre^ER^ mice at 50°C and 52°C, as compared with that obtained before IVM treatment and those from wild type controls before and post IVM treatment (Figure 4e). Wild type mice treated with IVM did not show a difference in the latency relative to that before IVM treatment (Figure 4e). In addition, the threshold for mechanical sensation shown by the von Frey test was increased also in IVM-treated Rosa26-IVMR;Prrxl1-Cre^ER^ mice (Figure 4f). To know the persistence of IVM-induced silencing effect, the von Frey test was performed at later time points. A significant increase in paw withdrawal threshold was observed also at 2 d, but it returned to normal level afterwards (Figure 4f). These results indicate that somatosensation is reduced in IVM-treated Rosa26-IVMR;Prrxl1-Cre^ER^ mice and IVMR is functional in Rosa26-IVMR mice crossed with transgenic Cre mice.

**Figure 5:**
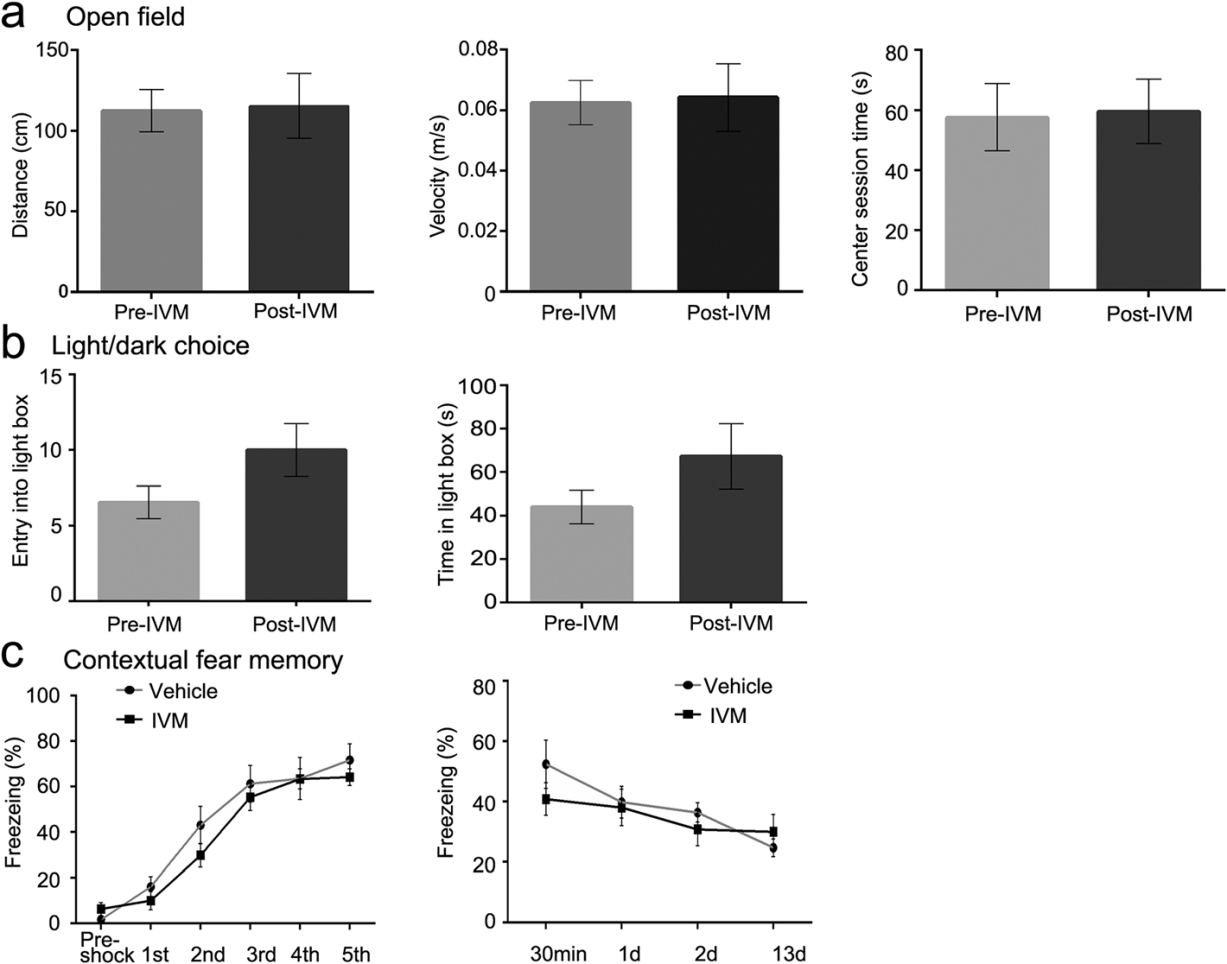
**Locomotor activity, anxiety-like behaviors and contextual fear memory are not altered by IVM treatment in wild type mice. (a)** The open field test shows that the total distance traveled, speed, and time spent in the center area are not different in the mice before and after IVM treatment. **(b)** The number of entering into and time spent in the light box are not significantly different in mice before- and after IVM treatment in the light/dark choice test. **(c)** Freezing time in the training and retrieval periods is not different between the vehicle- and IVM-treated mice in the contextual fear memory test.

Because a higher dose of IVM (e.g. 20 mg/kg, i.p.) induces tremors and partial paralysis in some mice [11,21], we examined the locomotor activity by open field test in wild type mice and Rosa26-IVMR mice treated with 10 mg/kg of IVM, the maximal concentration used in this study. There were no differences in distance traveled and speed recorded before and after IVM treatment in these mice (Figure 5a). Meanwhile, the time spent in the center area of the open field was similar before and after IVM treatment, reflecting no changes in the anxiety-like behaviors. This was further supported by the light/dark choice tests, in which the numbers of entering into and times spent in the light box also showed no differences before and after IVM treatment in the mice (Figure 5b). In addition, the contextual fear memory was similar between vehicle- and IVMR-treated wild type mice during both the training and retrieval periods (Figure 5c). We conclude that systemic IVM administration does not obviously affect locomotor activity, anxiety or contextual fear memory.

**Figure 6:**
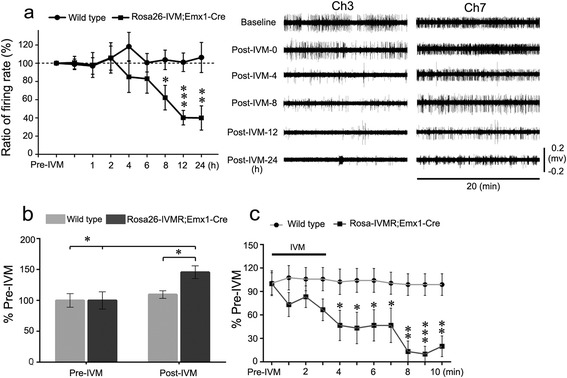
**IVM-induced reduction of neuron firing and excitability in Rosa26-IVMR mice. (a)** Neuron firing in the hippocampus of Rosa26-IVMR;Emx1-Cre mice is reduced after systemic administration of IVM. A significant reduction of neuron firing is present at 8 h, and it reaches the lowest level at 12 h and persists at 1 d. Recording was performed in free moving mice; 19 neurons from 4 wild type mice and 18 neurons from 2 Rosa26-IVMR;Emx1-Cre mice were included. *P < 0.05, **P < 0.01, ***P < 0.001, one-way ANOVA. Recordings from two reparative neurons in channel 3 and 7 are shown in the right panel. **(b)** Cortical neuron excitability shown by ratio of current threshold before and after IVM treatment is reduced in Rosa26-IVMR;Emx1-Cre mice after bath application of IVM, whereas it was unchanged in wild type mice. n = 7 for wild type, n = 8 for Rosa26-IVMR;Prrxl1-Cre^ER^ mice; *P < 0.05; 2-tailed Student’s t-test. **(c)** Time course of action potential frequency initiated by injection of current (450 pA, 400 ms) after bath application of IVM (10 nM, 3 min). Action potential frequency was normalized to that obtained before IVM treatment for comparison. Note that the frequency of action potential decreased rapidly and dramatically after IVM treatment. n = 6 for wild type, n = 5 for Rosa26-IVMR;Prrxl1-Cre^ER^ mice. *P < 0.05, **P < 0.01, ***P < 0.001; two-way repeated ANOVA test.

To examine if activation of IVMR inhibits neuronal firing, we performed *in vivo* recoding in free moving Rosa26-IVMR;Emx1-Cre mice by placing multiple electrodes in the hippocampus, which contained numerous neurons expressing IVMR (Figure 3f). After an intraperitoneal injection of IVM (10 mg/kg), a significant reduction of neuron firing rate was first observed at 8 h, and this reduction reached the lowest level at 12 h and maintained at 1 d (Figure 6a). However, IVM administration did not alter neuron firing in wild type mice (Figure 6a). Thus, a single IVM treatment leads to a long-lasting reduction of hippocampal neuron firing in Rosa26-IVMR;Emx1-Cre mice.

It takes time for IVM to reach the brain and to reduce neuronal firing via activation of IVMR after systemic administration of IVM. To know how fast IVM activates IVMR and reduces neuronal firing, we made electrophysiological recording from brain slices prepared from Rosa26-IVMR;Emx1-Cre mice. We found that the excitability of hippocampal neurons, shown by the minimal current injection (current threshold) evoking neuronal firing, was dramatically reduced in Rosa26-IVMR;Emx1-Cre mice, whereas it was unchanged in wild type controls after IVM treatment (Figure 6b). In addition, action potential frequency evoked by intracellular current injection (450 pA, 400 ms) were reduced substantially within minutes after bath application of IVM in Rosa26-IVMR;Emx1-Cre mice. By contrast, action potential frequency remained unchanged in wild type controls after IVM treatment (Figure 6c). These results demonstrate that IVM is able to lead to a reduction of neuronal firing by lowering its excitability in Rosa26-IVMR;Emx1-Cre mice.

## Discussion

Here we generated Rosa26-IVMR mice in which IVM reduced neuronal activity in a cell type-specific manner. IVMR expression was present in Rosa26-IVMR mice in the presence of virus-delivered Cre and when crossed with Cre lines, and reversible inhibition of neuronal activity was induced by systemic administration of IVM. This mouse line offers a new genetic tool for exploring the causal relationship between the silencing of specific neuronal circuits and behaviors.

Specific Cre-mediated IVMR expression in Rosa26-IVMR mice was confirmed by injecting of AAV2-Cre virus and crossing with several Cre lines (Figures 2 and 3). The ability of IVMR activation to silence neuronal activity was revealed by recording neuronal firing in free moving animals, and in brain slices (Figure 6) and by observing behavioral alteration (Figure 4). The data from apomorphine-induced rotation indicates that an obvious and steady behavioral alteration is observed at 12 h post systemic administration of IVM (Figure 4). This delay may reflect the timing for IVM to reach sufficient concentration in the brain for activating IVMR and inhibiting neuronal activity, because it takes only a few minutes for IVM to reduce neuronal excitability in brain slices (Figure 6). It is possible that intraventricular administration of IVM would achieve a faster onset of neuronal silencing if desired. Peak behavioral alteration was observed at 1 d, and decreased at 3 d but no longer existed at 5 d. This long-lasting silencing effect *in vivo* could be beneficial in experiments where continuous silencing is required during extended behavioral performances. However, this could be a disadvantage in some experiments requiring fast recovery. This long-lasting effect is consistent with the published data concerning the property of IVM-gated GluClαβ, and it may be caused by slow ligand clearance/metabolism and dissociation of IVM from the channels [11].

We examined the dose-dependent effect of IVM in apomorphine-induced rotation. IVM at 5 and 10 mg/kg led to similar rotational behavior at 12 h, but 10 mg/kg of IVM resulted in a longer period of rotational behavior compared with 5 mg/kg (1.5 d vs 12 h; Figure 4). Mechanical sensation shown by the von Frey test also indicated that 10 mg/kg of IVM led to a marked increase in the paw withdrawal threshold by 2 d after IVM administration. Together with other behavioral data, we have shown that both 5 and 10 mg/kg IVM is sufficient to inhibit neuronal activity *in vivo*, and that 5 mg/kg can be used if a shorter period of silencing is desired.

The inducible, reversible and re-activatable properties of the IVMR offer a unique advantage for designing experiments. The possible relationship between neuron circuit activity and behaviors can be verified repeatedly in the same mice, pre-IVM, post-IVM (phenotype), recovery and post-IVM (restore phenotype). Meanwhile, other methods of manipulating neuron activity [1–4] can be applied in the second cycle of neuronal silencing to clearly dissect the causal link between the specific neuronal circuit and behavior.

IVM is a toxic drug used for treatment of animals with infection of arthropod and parasitic nematode. The concentrations required for silencing neuron is between 5-10 mg/kg, which is higher than that used in anti-parasitic treatment. Chronic administration of IVM in the drinking water does not affect general health, body weight, motor coordination, swimming behavior, or spatial learning in mice [22]. Intraperitoneal injection of 20 mg/kg IVM and above induced tremors and partial paralysis in some mice, but this and a previous study [11] did not observe this adverse effect on locomotion when 5-10 mg/kg IVM was injected intraperitoneally. Additionally, IVM reduced alcohol intake and preference in mice [23], and 20 mM IVM could lead to maximal activation of GABA receptors expressed in HEK 293 cells [24]. Although we did not observe an obvious alteration in anxiety-like behaviors and contextual fear memory (Figure 5), it would be advisable to examine the adverse effects of IVM when Rosa26-IVMR mice were crossed with other mouse lines with abnormal GABA system or in cases of combination use of other chemicals/drugs that may affect clearance/metabolism of IVM.

## Materials and methods

### Generation of Rosa26-IVMR mice

Targeting constructs were generated using molecular cloning approach. Briefly, to target the Rosa26 locus, a cassette containing the IVMR-2A-GFP gene was constructed [12,14]. This cassette was cloned into a Rosa-CAG targeting vector [25], in which dtTomato was replaced with IVMR-2A-GFP gene to generate the final IVMR knock-in vector (Figure 1a). The final targeting vectors contained 5′and 3′ homology arms of 1.1 kb and 4.3 kb, as well as a PGK-DTA cassette for negative selection. The targeting vectors were linearized and transfected into the 129/B6 F1 hybrid ES cell line G442 using an Amaxa electroporator. G418-resistant ES clones were screened by PCR and sequencing combined analysis. PCR primers were designed according to the expected insertion pattern. The 5′ arm forward primer was designed according to the wild type sequences of the up-stream of the insertion site (5′-CGCGGAACTCCATATATGGGCTATG-3′), and the reverse primer was designed according to the insertion sequences down-stream of the insertion site (5′-GGCTCCTCAGAGAGCCTCGGCTAG-3′). Similarly, the 3′ arm forward primer was designed according to the inserted NeoR gene (5′-AGACCATTCTCAGTGGCTCAACAAC-3′) and the reverse primer was designed according to the wild type sequences of the down-stream of the insertion site (5′-ATTCGGCTATGACTGGGCACAACA-3′). Positive ES clones were injected into C57BL/6 J blastocysts to obtain chimeric mice that were bred with C57BL/6 J mice to obtain germline transmission.

### Animals

Adult (2-4 month old) Rosa26-IVMR mice were used. All animal care and experimental protocols were reviewed and approved by the Animal Research Committee of Tongji University School of Medicine, China.

Cre-mediated IVMR expression was examined in Rosa26-IVMR mice. The AAV2-Cre virus was prepared as reported previously [26,27], and was injected unilaterally into the striatum, Mice were sacrificed for detection of IVMR expression 14 d later, and this was used to show if IVMR expression could be induced by local delivery of Cre-expressing virus. Second, Rosa26-IVMR mice were crossed with Emx1-Cre [15], CamKII-Cre [13] and Prrxl1-Cre^ER^ [17] mice to show if IVMR expression could be activated by crossing with Cre line mice.

### Behavioral observation

Two behavioral paradigms were used to evaluate the effects of IVM-induced neuron silencing in Rosa26-IVMR mice. Two weeks after unilateral injection of AAV2-Cre virus into the striatum, IVM at doses of 2.5, 5.0 and 10.0 mg/kg (Jiangsu East China Bell Co., China) was administered by a single intraperitoneal injection, and apomorphine-induced rotation behaviors were examined at different time points. At each time point, apomorphine (5 mg/kg, i.p.; Sigma, USA)-treated mice were placed in Activity Monitor (Med Associates) for 20 min and rotation index was calculated by using EhtoVision XT8 software (Noldus, The Netherlands) as reported previously [11]. Rotation scores were calculated by subtracting contralateral from ipsilateral rotations and dividing by the total distance travelled (m), namely rotation scores = rotation numbers/distance (m). To examine if the IVMR could be re-activated, the rotation behavioral observation was performed again in the same mice 3 weeks after the initial IVM administration. Six cohorts of mice were used in this set of experiment to ensure at least a 1-day interval between two apomorphine treatments.

The second behavioral observation was performed in Rosa26-IVMR;Prrxl1-Cre^ER^ mice. Cre recombinase was induced by tamoxifen treatment as reported in our previous study [17]. Four weeks later somatosensory sensation was examined by both the von Frey test [28] and the tail immersion test (50°C and 52°C) at 1 d post a single administration of IVM (10 mg/kg; i.p.). In addition, to explore if IVM affects locomotor activity, wild type mice and Rosa26-IVMR mice with unilateral injection of AAV2-Cre virus were placed in a plexiglass box (Med Associates) and locomotor activity was monitored over a 30-min period. Four to seven days later, the same mice were treated with IVM (10 mg/kg, i.p.), and locomotor activity was monitored again at 1d post IVM treatment. Furthermore, the light/dark choice test was used to examine if the anxiety-like behaviors were altered by IVM. These examinations were performed in the wild type mice. Mice were subjected to the test without IVM first, and 7-10 d later to the test again with IVM injection that was done 1d before the test commenced. The light/dark choice test apparatus consisted of a small dark chamber (30 cm × 20 cm × 30 cm) connected by an opening (5 cm × 7 cm) to a larger lit chamber (30 cm × 30 cm × 30 cm). A single mouse was initially placed in one corner of the dark chamber and the percentage of time spent in the lit chamber was measured over 5 min.

Contextual fear conditioning was performed as described in our previous study [29]. FreezeFrame and FreezeView software systems (Coulbourn Instruments, USA) were used to record and analyze freezing behaviors. Wild type mice treated with vehicle or IVM (10 mg/kg, i.p.) were given five foot shocks (0.7 mA, 2 s) at 2 min intervals during which the mice were able to move freely. The percentage of freezing time was measured during each inter-shock interval. Thirty minutes, 1 d, 2 d and 13 d later, the mice were placed back in the same box for 11 min without receiving any foot shocks, and freezing time was measured to test contextual fear memory.

### Immunohistochemistry and *in situ* hybridization

Mice were anesthetized deeply with sodium pentobarbital and perfused through the ascending aorta with 0.01 M phosphate buffered saline (PBS; pH 7.4) followed by 4% paraformaldehyde in 0.1 M phosphate buffer (pH 7.4). After cryoprotection with 30% sucrose in PBS, samples were cut transversely into 40 μm-thick sections on a cryostat. The following primary antibodies were used: mouse anti-NeuN antibody (1:1000; Chemicon, USA), rabbit anti-human glycine receptor antibody (1:200; Millipore, USA) and rabbit anti-GFP antibody (1:1000; Invitrogen, USA). Sections were incubated with the anti-GFP antibody overnight at 4°C, and then with biotinylated goat anti-rabbit secondary antibody (1:500; Vector Laboratories, USA) for 2 h at room temperature, followed by incubation with the avidin-biotin complex (Vector Laboratories). Immunoreactive signals were detected by using 3,3′-diaminobenzidinetetrahydro-chloride (Sigma, USA) as a chromogen. For double immunofluorescence, sections were incubated with a mixture of rabbit anti-human glycine receptor and mouse anti-NeuN antibodies, followed by a mixture of Alexa Fluor 488-conjugated anti-mouse (1:500; Invitrogen, USA) and Cy3-labeled anti-rabbit IgG (1:500; Jackson ImmunoResearch, USA). Sections were observed under a Nikon fluorescence microscope equipped with a Nikon Coolpix digital camera (DS-Ri1; Japan) or using laser confocal microscope (TCS SP5 II; Leica, Germany).

Antisense digoxigenin-labeled RNA probes for the human Glycine receptor were synthesized and *in situ* hybridization was performed as described previously [18]. All images were made into figures using Adobe Photoshop (Adobe Systems Incorporated, USA) and only minor adjustments to the contrast and brightness settings were applied if necessary.

### *In vivo* electrophysiological recording

For extracellular recording of neuron firing, adult free moving Rosa26-IVMR;Emx1-Cre mice were used. The recording electrode bundle contains 32 single wires (California FineWire, USA). Under anesthesia with sodium pentobarbital (8 mg/kg, i.p.), and the tips of the electrodes were positioned to encounter dorsal CA1 region, according to the specific local field potential and neuronal activity patterns. After stabilization with dental cement, mice were allowed to recover for 7 d. Principal component analysis was used to extract defining features from the spike wave shapes, and units were isolated using spike sorting software (Offline Sorter 3.0, USA). Stable units must be well isolated before experiments. During experiments, we recorded the activity of individual neurons during freely moving states. Each mouse underwent numerous recording sessions through the whole experiment. Firing frequency of individual neuron after IVM treatment was normalized to that before IVM treatment for comparison.

### *In vitro* electrophysiological recording

For recording in brain slice, 3-6 weeks old Rosa26-IVMR;Emx1-Cre mice were used. Transverse brain slices (350 μm) were cut on a vibrating microslicer (Leica VT1200, Germany). Whole-cell patch-clamp recordings were performed after the brain slices were incubated for 1 h in external artificial CSF (in mM: NaCl 117, KCl 3.6, CaCl_2_ 2.5, MgCl_2_ 1.2, NaH_2_PO_4_ 1.2, NaHCO_3_ 25 and glucose 11) which was bubbled continuously with carbogen (95%O_2_/5%CO_2_). Recording pipettes with resistances of 3–5 MΩ were pulled from borosilicate glass (P-97; Sutter Instruments, USA) and filled with a solution of (in mM) potassium gluconate 135, KCl 5, CaCl_2_ 0.5, MgCl_2_ 2, EGTA 5, HEPES 5 and ATP-Mg 5. Evoked action potential were measured in layer II-III cortical neurons in current clamp mode. The signals were amplified with an Axopatch 700B amplifier (Molecular Devices, USA), filtered at 2 kHz, and digitized at 5 kHz. The data were stored and analysed with a personal computer using software of pCLAMP 10. The minimal injected current for evoking action potential (current threshold) were measured first, and was measured again after bath application of IVM (10 nM; Sigma). Current threshold obtained after IVM treatment was normalized to that before IVM treatment for statistical comparison. In addition, time course of action potential frequency initiated by injection of current (450 pA, 400 ms) was examined as well.

### Quantification and statistics

Data are expressed as mean ± SEM. Differences between two groups were compared using 2-tailed Student’s t-test. One way ANOVA followed by a Fisher post hoc test to evaluate the differences of rotational scores among multiple concentration groups in 12 h and 24 h, respectively. Two way repeated ANOVA was used for comparison of rotational score between 10 mg/kg and 5 mg/kg IVM groups in various time points and two different genotypes between before and after IVM treatment. All the data were analyzed using PASW Statistics 18. The criterion for statistical significance was P < 0.05.

## Additional files

Additional file 1: Figure S1. Double immunostaining showing Cre expression in the striatum of wild type mice injected with AAV2-Cre virus. AAV2-Cre virus contains GFP expressing sequence. Arrows indicate neurons expressing both GFP and Cre immunoreactivity. Scale bars = 150 μm (a-a”) and 50 μm (b-b”). Additional file 2: Figure S2. Time course of apomorphine-induced rotations in Rosa26-IVMR mice treated with 5 mg/kg IVM. Obvious rotation behaviors are present at 5 h and 9 h post IVM treatment, while rotation scores at these two time points varied greatly relative to those at later time points. n = 9.

## Abbreviations

CA1: CA1 region of hippocampus
DG: Dentate gyrus
DH: Spinal dorsal horn
dl: Dorsal fasciculus
IVM: Ivermectin
IVMR: Ivermectin-gated silencing receptor
lf: Lateral fasciculus
I-VI: Cortical layers
