# Supplementary figures and images for: A mouse line for inducible and reversible silencing of specific neurons
Source: Mol Brain. 2014 Sep 18;7:68. doi: 10.1186/s13041-014-0068-8 (PMC4177062; doi:10.1186/s13041-014-0068-8)

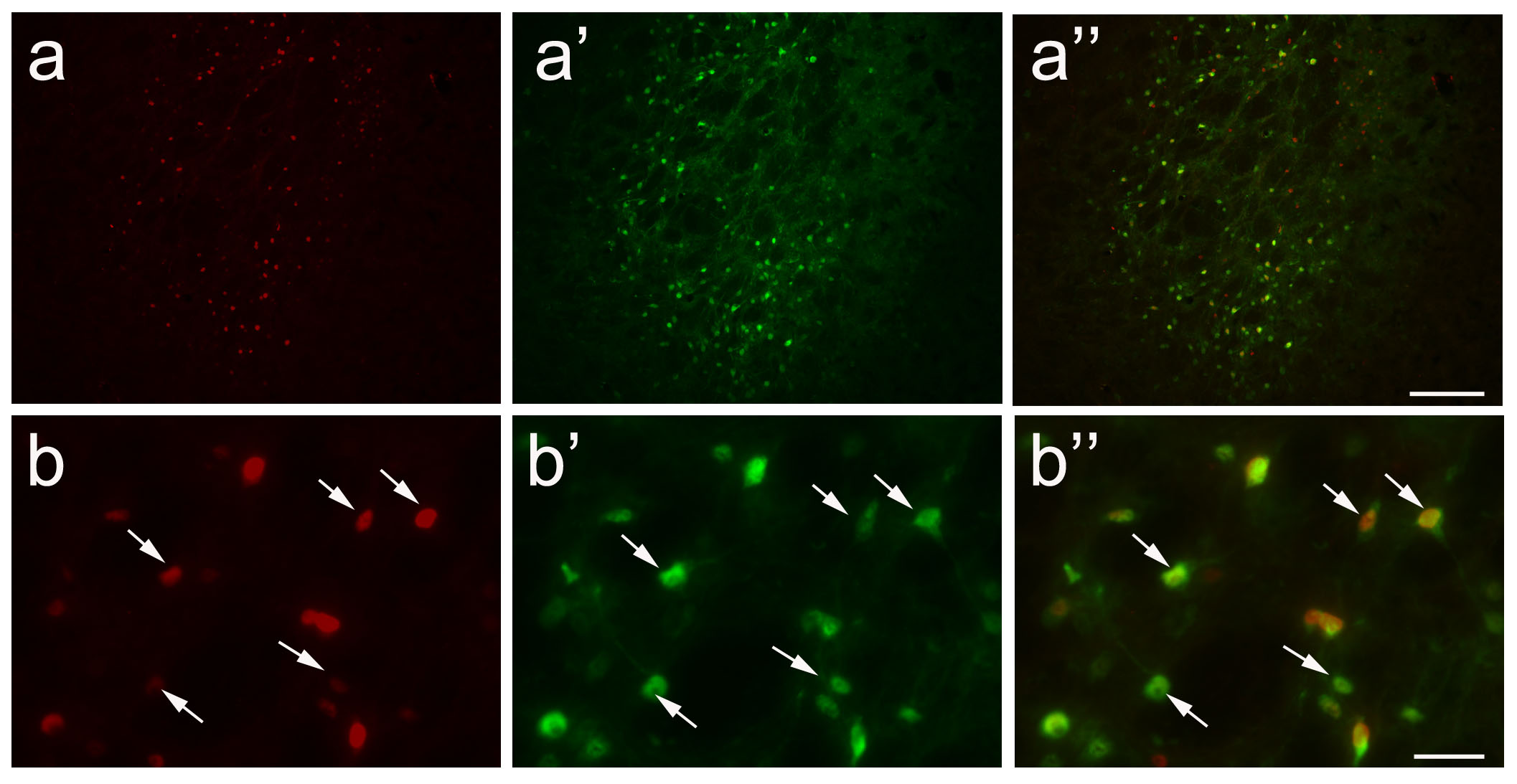

Supplement: Additional file 1: Figure S1. — Double immunostaining showing Cre expression in the striatum of wild type mice injected with AAV2-Cre virus. AAV2-Cre virus contains GFP expressing sequence. Arrows indicate neurons expressing both GFP and Cre immunoreactivity. Scale bars = 150 μm (a-a”) and 50 μm (b-b”). [file 13041_2014_68_MOESM1_ESM.jpeg]

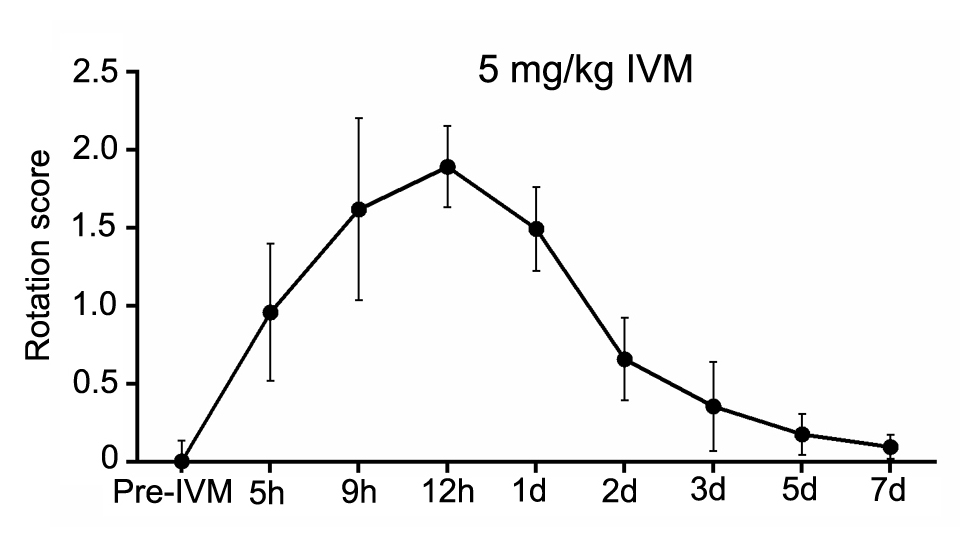

Supplement: Additional file 2: Figure S2. — Time course of apomorphine-induced rotations in Rosa26-IVMR mice treated with 5 mg/kg IVM. Obvious rotation behaviors are present at 5 h and 9 h post IVM treatment, while rotation scores at these two time points varied greatly relative to those at later time points. n = 9. [file 13041_2014_68_MOESM2_ESM.jpeg]
